# Age Differences in Anticipated Use of Virtual Healthcare Services After the Pandemic

**DOI:** 10.1093/geroni/igab046.3558

**Published:** 2021-12-17

**Authors:** Matthew Murphy, William Hills, Karen Hills

**Affiliations:** 1 Coastal Carolina University, Conway, South Carolina, United States; 2 Beaufort Jasper Hampton Comprehensive Health Services, Conway, South Carolina, United States

## Abstract

Healthcare has undergone a significant transformation during the pandemic, with virtual services being rapidly developed and implemented to keep pace with societal needs. This study documented this change in healthcare by examining access and use of video-based, virtual service use before and during the pandemic. Participants for the study (n = 685) included three groups, including retirement-aged persons, middle-aged adults, and traditional college-aged students. Measures for the study included access to and utilization of physical and mental health services, satisfaction with services accessed, and anticipated access and use of virtual services following the pandemic. Results showed that most participants (94.2%) believed that virtual healthcare would persist after the pandemic; three-quarters of adults (75.2%) but only half of college-aged (52.8%) and retirement-aged (57.6%) participants anticipated using virtual healthcare in the future. Prior use and satisfaction with virtual healthcare services mediated anticipated future use for retirement-aged participants (p < .001), but only satisfaction with virtual healthcare was a marginal predictor for college-aged participants (p = .051), and neither were predictors for adult-aged participants. These results support that people believe virtual healthcare will persist after the end of the pandemic, but that there are age-related differences in who anticipates using these services in the future, and which factors will make the most difference in attracting clients. These differences can impact how healthcare providers market and develop further tele-health services to increase the likelihood of use by retirement-aged participants, and suggests that client satisfaction is a key mediator for different age groups.

